# Growing and Etching MoS_2_ on Carbon Nanotube Film for Enhanced Electrochemical Performance

**DOI:** 10.3390/molecules21101318

**Published:** 2016-09-30

**Authors:** Weiyu Xu, Qi Fang, Daobin Liu, Ke Zhang, Muhammad Habib, Chuanqiang Wu, Xusheng Zheng, Hengjie Liu, Shuangming Chen, Li Song

**Affiliations:** National Synchrotron Radiation Laboratory, CAS Center for Excellence in Nanoscience, University of Science and Technology of China, Hefei 230029, China; xuweiyu@mail.ustc.edu.cn (W.X.); fq4311@mail.ustc.edu.cn (Q.F.); ldbin@mail.ustc.edu.cn (D.L.); zk2009@mail.ustc.edu.cn (K.Z.); mhabib@mail.ustc.edu.cn (M.H.); wucq@mail.ustc.edu.cn (C.W.); zxs@ustc.edu.cn (X.Z.); lhjbh@mail.ustc.edu.cn (H.L.); csmp@ustc.edu.cn (S.C.)

**Keywords:** carbon nanotube, molybdenum disulfide, CVD, acid etching, electrochemical performance

## Abstract

In this work we directly synthesized molybdenum disulfide (MoS_2_) nanosheets on carbon nanotube film (MoS_2_@CNT) via a two-step chemical vapor deposition method (CVD). By etching the obtained MoS_2_@CNT into 10% wt HNO_3_, the morphology of MoS_2_ decorated on CNT bundles was modulated, resulting in more catalytic active MoS_2_ edges being exposed for significantly enhanced electrochemical performance. Our results revealed that an 8 h acid etching sample exhibited the best performance for the oxygen evolution reaction, i.e., the current density reached 10 mA/cm^2^ under 375 mV over-potential, and the tafel slope was as low as 94 mV/dec. The enhanced behavior was mainly originated from the more catalytic sites in MoS_2_ induced by the acid etching treatment and the higher conductivity from the supporting CNT films. Our study provides a new route to produce two-dimensional layers on CNT films with tunable morphology, and thus may open a window for exploring its promising applications in the fields of catalytic-, electronic-, and electrochemical-related fields.

## 1. Introduction

Growing industrialization and the immense use of energy which is usually obtained from fossil fuels create a significant harmful impact on the climate and human health. Due to rapid exhaustion of fossil energy and environmental concerns, people are now exerting enormous efforts to develop renewable energy sources such as solar energy [1,2,3,4], wind power [5,6,7] and bioenergy [8,9,10,11,12], etc. Hydrogen generated by splitting water has a great potential as an ideal future energy source because of its high energy density and lack of toxicity features. The water splitting process can be divided into two half-processes [13], the hydrogen evolution reaction (HER) and the oxygen evolution reaction (OER). Because of the energy and dynamic barrier, to drive the reaction on, a potential higher than 1.23 V is essential. Between these two half-processes, OER is the bottleneck because of the multi-proton transfer process [14]. Introduction of a catalyst can significantly reduce this over-potential, thus resulting in improved energy conversion efficiency. Iridium oxidate is the best catalyst for OER [13] but its practical uses suffers from high price and low earth abundance, while transition metal compounds can overcome these two disadvantages and can be a good substitution to catalyze the OER reaction according to the volcano plot [13].

Molybdenum disulfide (MoS_2_), a typical layered transition metal dichalcogenide (TMDC), which has a 1.29 eV indirect bandgap in the bulk state and a 1.8 eV direct bandgap in the monolayer state [15], is not only the ideal candidate for future semiconductor materials [15,16,17], but also a suitable catalyst in the electrochemical catalytic field [18,19] and a lithium battery anode material [20,21,22]. There are three shortcomings which hinder the practical use of MoS_2_ as a catalyst in the water splitting process. First, as a typical semiconductor, MoS_2_ has quite poor conductivity, which causes poor electron transfer ability. Second, because of the tension strength released during the catalyzing process, catalysts are not stable. To solve these two problems, composites containing MoS_2_ with a substrate such as reduced graphene oxide [23], carbon nanotubes [20], or nickel foam [24], etc., have been developed and tested. Such substrates provide an excellent electron conducting network and stabilize the MoS_2_ in the network. Third, bulk MoS_2_ has few active sites. Reducing the size of the MoS_2_ is a feasible method to increase the active sites. There are a lot of research works focusing on the synthesis of MoS_2_ nano-flowers [25], nano-sheets [26] and nano-rods [27] or introducing defects such as unsaturated sulfur [28] to enhance the catalyzing performance. Most of those works are based on the hydrothermal method.

In our previous work, we synthesized a non-woven carbon nanotube film (CNT film) with a good mechanical property and high conductivity [28]. Herein, we employed such a CNT film as a supporting material, and used a developed chemical vapor deposition method to directly grow MoS_2_ on the CNT film for preparing hybridized structures (MoS_2_@CNT). As follows, an acid treatment with 10% wt HNO_3_ was also supposed to reduce the thickness and tune the morphology of the obtained MoS_2_ nanosheets in order to create more active catalytic sites in the MoS_2_ being exposed. Meanwhile, the entangled nanotube bundles among the CNT film can further accelerate the transport of electrons. Therefore, such deliberate processes will open up a new way for producing high-performance synergistic hybrid materials for enhanced electrochemical performance.

## 2. Results

### 2.1. Synthesis and Characterization of MoS_2_@CNT Composite

**Figure 1:**
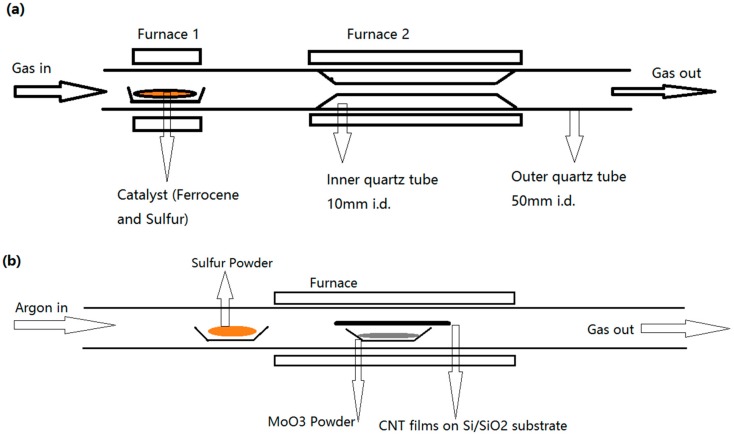
Schematic route of the CVD configuration used to grow: (**a**) CNT film; and (**b**) MoS_2_@CNT hybrids.

Our CVD configuration that has been used to synthesize CNT film and MoS_2_@CNT [29] is shown in Figure 1a,b. The detailed synthesis process can be found in our previous work [30] and is also described in the following Materials and Methods section.

**Figure 2:**
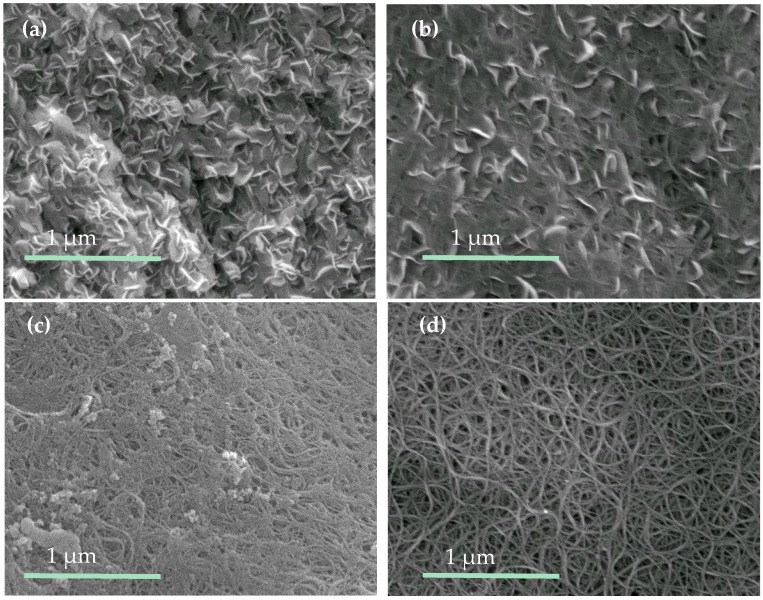
SEM surface morphology of MoS_2_@CNT composite: (**a**) pristine sample and (**b**–**d**) treated with 10% wt. HNO_3_ for 2 h, 8 h, and 12 h.

**Figure 3:**
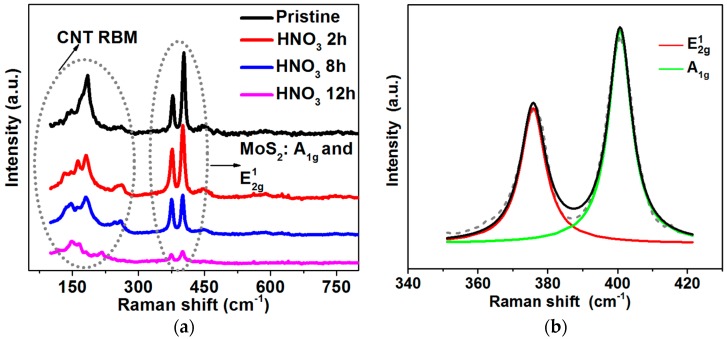
Raman spectrum of MoS_2_@CNT: (**a**) Pristine and treated samples with HNO_3_ for 2 h, 8 h and 12 h. (**b**) Deconvolution results of pristine MoS_2_@CNT for $E_{2g}^{1}$ and $A_{1g}$ modes.

**Figure 4:**
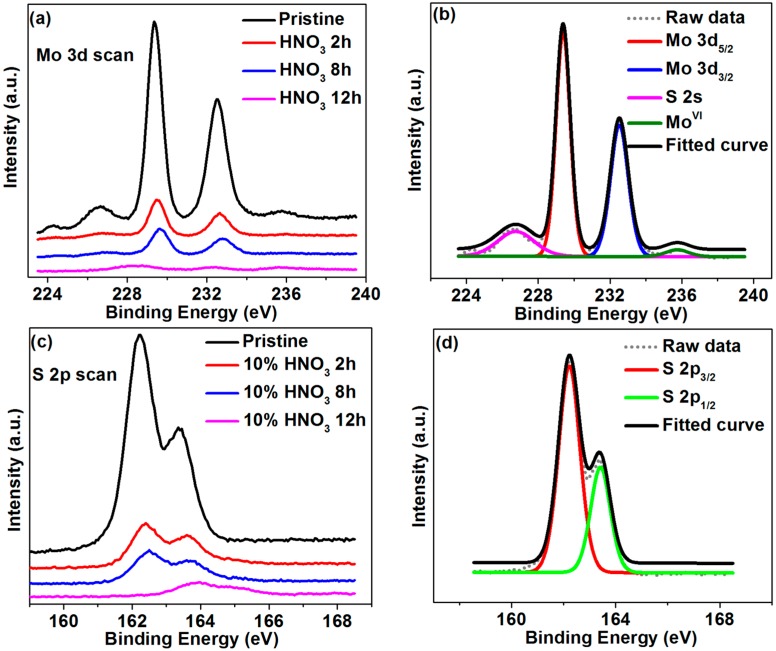
XPS results of different samples: (**a**,**c**) Mo 3d and S 2p curve comparison among different durations of acid-treated (pristine, 2 h, 8 h, 12 h) MoS_2_@CNT, and (**b**,**d**) pristine MoS_2_@CNT Mo 3d and S 2p deconvolution.

Scanning electronic microscopy (SEM) characterization results are shown in Figure 2a–d. In the as-grown MoS_2_@CNT sample, MoS_2_ with a pillar-like structure stacks layer by layer, thus forming thick coverage on top of the CNT film. The thick MoS_2_ layer has poor conductivity and fewer active sites for catalysis, which decreased the electrochemical performance. To reduce the thickness of the MoS_2_ cover, as-grown MoS_2_@CNT samples were treated with 10% wt. HNO_3_ for different durations. By controlling the treatment duration, we suggest that the thickness and morphology of MoS_2_ can be selectively modulated; hence, the electrochemical catalysis performance will be subsequently optimized. The experimental results for different durations of acid etching are shown in Figure 2b–d. After 2 h treatment the MoS_2_ layer thickness has been significantly reduced, thus exposing the network structure of the CNT film which is illustrated in Figure 2b. By extending the acid etching duration to 8 h (Figure 2c), the pillar structure can be further dissolved and the MoS_2_ coverage area is considerably diminished. When we increased the acid etching duration to 12 h, most of the MoS_2_ coverage was removed from the CNT film surface and the clear nanotube network structure remained, as shown in Figure 2d. In fact, there is still MoS_2_ retention detected from the Raman spectroscopy in Figure 3a and the X-ray photoelectron spectrum (XPS) in Figure 4a,c.

The Raman spectrum is a very useful tool to identify the existence of MoS_2_ [31]. Figure 3a shows the typical Raman spectra of pristine MoS_2_@CNT and acid-treated MoS_2_@CNT with different treatment durations. From the Raman spectra it can be observed that there are two main peak regions; one is at around 100–300 cm^−1^ which corresponds to the radial breathing mode (RBM) of single-wall carbon nanotubes [30], while the other is at 300–450 cm^−1^ and is related to the MoS_2_. The zoom-in and deconvolution results in Figure 3b clearly show two distinguishable vibration modes of MoS_2_, the in-plane vibration mode $E_{2g}^{1}$ (~383 cm^−1^ for bulk MoS_2_) and the out-of-plane vibration mode $A_{1g}$ (~405 cm^−1^ for bulk MoS_2_). The intensity of the $E_{2g}^{1}$ and $A_{1g}$ peaks was decreased with the increase of the HNO_3_ etching duration. Although the intensity of those two vibration modes was significantly decreased for samples treated for 12 h, their detection in the spectra reveals the existence of MoS_2_ at the CNT surface which is also confirmed by the following XPS results.

Figure 4 shows the XPS characterization of different-acid-etching-duration samples. The calibration was done by referencing all XPS spectra to the C (1s) peak in Figure S3. In the as-grown MoS_2_@CNT samples, typical Mo 3d_5/2_ (at 229.4 eV), Mo 3d_3/2_ (at 232.5 eV), together with highly oxidized Mo (MoO_3_ at 235.8 eV), can be seen in the deconvoluted curves [24,28]. It is worth noting that the atomic ratio of Mo and S in the as-grown MoS_2_@CNT is 1:2.14, calculated from Figure 4b,d.

With the increase of the HNO_3_ etching duration, Mo 3d and S 2p bonding became smaller and broadened, which means that the atomic ratios of these atoms were decreased. Meanwhile, the highly oxidized Mo peak disappeared, referring to the fact that MoO_3_ was reacted and dissolved in the HNO_3_ solution. Those phenomena showed that MoS_2_ has been etched away during acid treatment, which was also proved by the above SEM characterization (Figure 2b–d).

The surface Molybdenum (Mo) and carbon (C) atomic ratio calculated from Figure 4a and Figure S3 with an integrated peak surface area and sensitive factor correlation [32] can refer to the relative MoS_2_ component on the CNT film substrate surface, as shown in Table 1. With the increase of the HNO_3_ etching duration, MoS_2_ on the CNT film surface reduces significantly.

### 2.2. Electrochemical Performance Test of MoS_2_@CNT Composite

**Figure 5:**
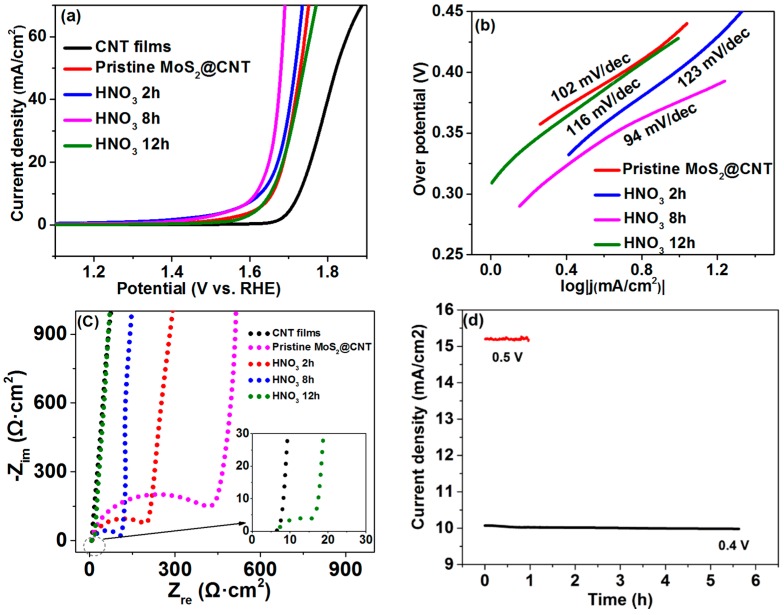
Electrochemical performances of different acid etching MoS_2_@CNT: (**a**) LSV curves of CNT film, pristine MoS_2_@CNT, MoS_2_@CNT treated with HNO_3_ for 2 h, 8 h and 12 h; and (**b**) corresponding tafel slope; (**c**) Impedance curves of pristine MoS_2_@CNT, MoS_2_@CNT treated with HNO_3_ for 2 h, 8 h and 12 h; (**d**) Current-time plot of the 8 h HNO_3_ etching duration MoS_2_@CNT.

The electrochemical measurements of MoS_2_@CNT have been carried out with 1 M NaOH as an electrolyte and Hg/HgO as a reference electrode. Our results are shown in Figure 5. All data has been converted to a reversible hydrogen electrode (RHE). As shown in Figure 5a, the linear sweep voltammetry (LSV) results have been corrected by IR compensate.

Obviously, the MoS_2_@CNT exhibited a better OER catalyzing performance as compared to the CNT film. With the increase of the HNO_3_ etching duration, the MoS_2_@CNT catalyzing performance showed significant improvement, but after a certain point, the performance was decreased. The required over-potential when the current density reaches 10 mA/cm^2^ is 504 mV, 435 mV, 404 mV, 375 mV and 428 mV for CNT film, pristine MoS_2_@CNT, and MoS_2_@CNT treated with HNO_3_ for 2 h, 8 h and 12 h.

From Figure 5b, with the increase of the acid etching duration, the tafel slope of MoS_2_@CNT showed clear changes. In particular, the 8 h acid-treated sample had the smallest tafel slope, suggesting the best dynamic process among those etching samples. Impedance tests were performed on the samples, as shown in Figure 5c. Pristine MoS_2_@CNT showed a 400 Ω electron transfer resistance (R_ct_). With the increase of the acid etching duration, R_ct_ decreased gradually, as expected. The inserted curve of Figure 5c revealed that the CNT film has a very small R_ct_ (~5 Ω), which can promote the catalyzing performance. Based on the above data, Figure 5d showed the catalytic performance stability of the HNO_3_ etching duration MoS_2_@CNT samples. The catalytic performance showed a slight decrease after a 6 h test under a 0.4 V over-potential. When increasing this over-potential to 0.5 V, the current density can reach 15 mA/cm. However, after a 1 h test, the current density dropped rapidly, which means the catalytic stability decreased.

## 3. Discussion

The pristine MoS_2_@CNT has a fairly thick MoS_2_ layer, thus it has fewer active sites (Figure 2a) and poor electron transfer ability (Figure 5c). When treated with 10% wt. HNO_3_, the MoS_2_ reacted with HNO_3_ and formed MoO_3_, and then MoO_3_ further reacted with HNO_3_ and was dissolved into the solution. The possible mechanism can be elucidated with the following chemical process [33].

(1) $${\mathrm{MoS}}_{2}\;\overset{{\mathrm{HNO}}_{3}}{\to }\;{\mathrm{MoO}}_{3}+{\mathrm{SO}}_{2}↑$$

(2) $${\mathrm{MoO}}_{3}\;\overset{{\mathrm{HNO}}_{3}}{\to }\;H_{2}{\mathrm{MoO}}_{4}$$

**Figure 6:**
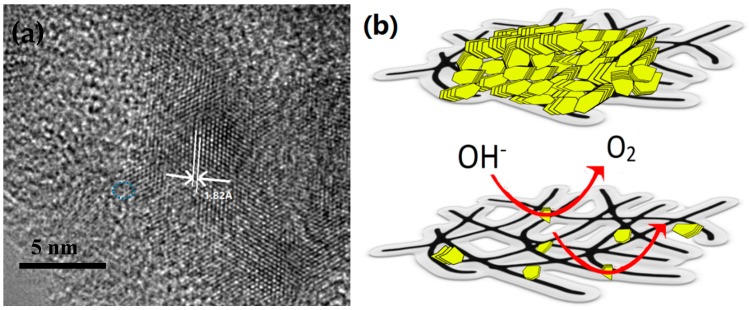
TEM images of MoS_2_@CNT: (**a**) high magnification image of MoS_2_@CNT and 1.82 Å is referred to the 2H-MoS_2_ (105) plane [34]. (**b**) Schematic illustration of: top, pristine MoS_2_@CNT and bottom, etching MoS_2_@CNT OER process. Black network refers to CNT film and yellow sheets refer to MoS_2_.

After etching with acid for different time durations, the MoS_2_ layer was etched gradually; also the layer integrity was destroyed, as shown in the SEM results (Figure 2b,c), which was further proved by the transmission electron microscopy (TEM) results (Figure 6a). More MoS_2_ edges were exposed which resulted in more active sites (Figure 6b). Meanwhile, the reduction of the layer thickness can guarantee sufficient contact between the MoS_2_ and CNT film; hence, the electron conductivity could be greatly improved. In fact, a further increased acid etching duration (more than 8 h) can further enhance the electron transfer ability, but the active sites decreased significantly, resulting in a poor catalyzing performance (Figure 2d and Figure 5a).

## 4. Materials and Methods

MoO_3_ (99.5%) was purchased from Alfa Aesar (Yingong RD No. 229, Fengxian Chemical District, Shanghai, China), The other reagents were purchased from Sinopharm Chemical Reagent Co. Ltd. (Ningbo RD No. 22, Shanghai, China): Sulfur (99.999%), Ferrocene (98%), Ethanol (AR), HCl (37%, AR), and HNO_3_ (66%, AR). All chemical reagents have been purchased from company and used without further purification.

### 4.1. Synthesis of CNT Film

Carbon nanotube film was synthesized by a floating CVD method as reported before [30]. The configuration is shown in Figure 1a. A special designed 50 mm diameter quartz tube with a 10 mm diameter inner tube was placed in furnace (MTK Co. Ltd., Hefei, China) and extra heating belt was set to heat the catalyst (16:1 molar ratio Ferrocene:S).

CNT film has been prepared by the following temperature process: main heating zone was heated to 1000 °C with a 30 °C/min ramping rate and a 200 sccm argon airflow was introduced as protective atmosphere. Next, temperature was increased to 1100 °C with 10 °C/min rate and at the same time second furnace was set to heat the catalyst at 90 °C. On reaching to 1100 °C, argon flow was increased to 1000 sccm and mixed with 3 sccm methane flow. After two hours of growth time, furnace was stopped and cooled to room temperature naturally. The as prepared CNT film has a high stretchable big size as shown in Figure S1.

The as-grown CNT film was first oxidized at 300~400 °C for 12~24 h to remove amorphous carbon, then immersed in 37% HCl for seven days to get rid of iron particles induced by ferrocene. After these purification steps, a clean random carbon nanotube network could be seen as shown in Figure S2. The purified CNT film was stored in ethanol.

### 4.2. Synthesis of MoS_2_@CNT

To get a uniform MoS_2_ layer on CNT film, first extended CNT film in Distilled Water and then transfer on Si/SiO_2_ substrate. After drying in oven at 90 °C, CNT film was placed upside down on a ceramic boat where deposed 14 mg MoO_3_. As shown in Figure 1b, ceramic boat was place in the middle of furnace, and at the edge of furnace hot zone, another boat with 120 mg sulfur powder was placed.

The growing process can be described as following steps. First 1000 sccm argon flow was introduced to get rid of air remaining in quartz tube. After 10 min argon rinsing, increased temperature to 750 °C with a 50 °C/min ramping rate, and MoS_2_ growth time lasted for 2 min at this temperature. After growing process was finished, to ensure good MoS_2_ structure, furnace has been opened to cool the quartz tube rapidly.

As-grown MoS_2_@CNT was treated in 10% wt. HNO_3_ for different times to etch MoS_2_ layer. After treated with HNO_3_, MoS_2_@CNT can be peeled off from Si/SiO_2_ substrate for next characterizations and testing steps.

### 4.3. Characterizations

Scanning Electron Microscopy (SEM). A field emission scanning electron microscope (SEM 15 kV, JEOL, JSM-6700F, Tokyo, Japan).

X-ray Photoelectron Spectroscopy (XPS, AXIS-HIS, Kratos Analytical, Hadano, Japan). The Photoemission Endstation at the BL10B beamline in the National Synchrotron Radiation Laboratory (NSRL) in Hefei, China.

Transmission electron microscopy (TEM). A field emission transmission electron microscope (TEM 200 kV, JEOL, JEM-2100F, Tokyo, Japan).

X-ray Diffraction (XRD, Japanese Rigaku Company, Tokyo, Japan). A D8-Advance power diffractometer with a Cu-Kα radiation source (λ = 1.54178 Å).

Raman Spectra. A Horiba microscopic Raman spectrometer (XploRA, HORIBA, Ltd., Tokyo, Japan). The laser having wavelength 532 nm (~2.5 mW/cm^2^) over a range of 70–3000 cm^−1^.

### 4.4. Electrochemical Measurements

All the electrochemical measurements were carried out with an electrochemical workstation (CHI660D electrochemical workstation) in a standard three-electrode cell with Hg/HgO and Pt mesh as the reference electrode and counter electrode, respectively. A glassy carbon (GC) electrode (3 mm in diameter) acted as the working electrode. OER measurements were conducted in 1 M NaOH as the electrolyte, which was saturated with oxygen during the experiments. The LSVs were performed at a scanning rate of 5 mV/s. The EIS spectrum was carried out with a 5 mV perturbative potential. The amperometric i-t curve was carried out with the same test condition as LSV scan.

## 5. Conclusions

In summary, we have employed a purified non-woven CNT film as a supporting material for directly growing MoS_2_ on it with the CVD method. The obtained samples showed hybridized structures with thick MoS_2_ nanosheets decorated on the CNT bundles. By etching the obtained MoS_2_@CNT with 10% wt. HNO_3_, the morphology of MoS_2_ has been tuned to expose more catalytic active sites. Meanwhile, the contact between the MoS_2_ and CNT film became tighter; hence, the electron transfer ability has been improved. The synergistic effect subsequently enhanced the electrochemical performance for OER catalysis. This work shows a possibility to directly prepare CNT-based hybrids and provides a way to tune the electron structure of the hybrids with acid treatment. Considering the fact that CVD method can be easily conducted for industrial producing, this study with the CVD growth and acid-etching process may be quite essential for future CNT-based electrochemical and electronic applications.

## Figures and Tables

**Table 1:** Surface Mo and C atomic ratio of different HNO_3_ etching duration samples.

Atomic Ratio	Pristine	10% wt HNO_3_ 2 h	10% wt HNO_3_ 8 h	10% wt HNO_3_ 12 h
Surface Mo and C atomic ratio	1.555	0.073	0.047	0.009

## Supplementary Materials

Supplementary materials can be accessed at: http://www.mdpi.com/1420-3049/21/10/1318/s1.
