# Supplementary material for: Growing and Etching MoS2 on Carbon Nanotube Film for Enhanced Electrochemical Performance
Source: Molecules. 2016 Sep 30;21(10):1318. doi: 10.3390/molecules21101318 (PMC6274235; doi:10.3390/molecules21101318)
Supplement: Supplementary file 1 [file molecules-21-01318-s001.pdf]

Supplementary Materials: Growing and Etching MoS_2 on Carbon Nanotube Film for Enhanced Electrochemical Performance

Weiyu Xu, Qi Fang, Daobin Liu, Ke Zhang, Muhammad Habib, Chuanqiang Wu, Xusheng Zheng, Hengjie Liu, Shuangming Chen and Li Song

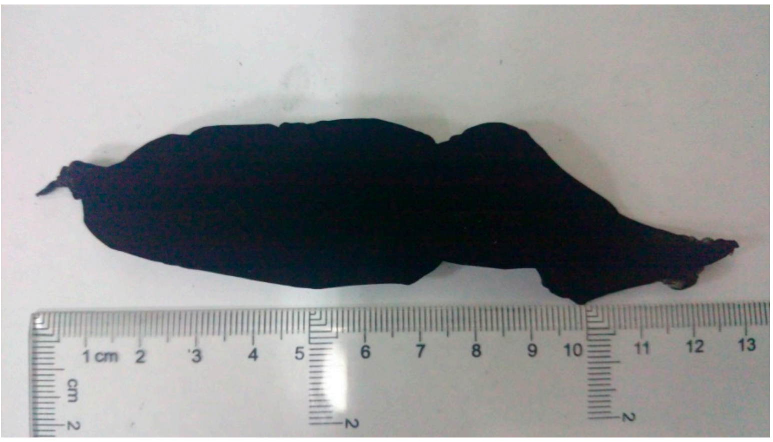

Figure S1. Optical image of as-grown CNT film. Size: 12 cm × 4 cm.

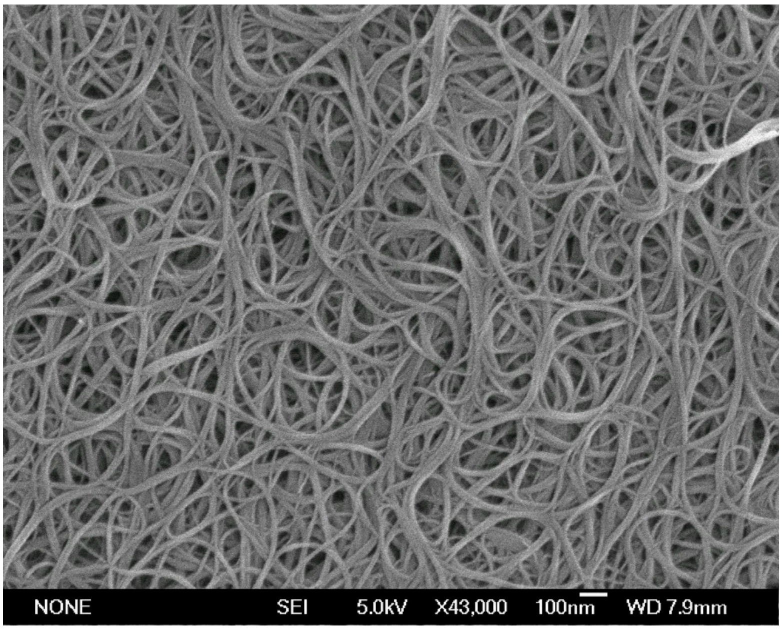

Figure S2. SEM characterization of purified CNT film.

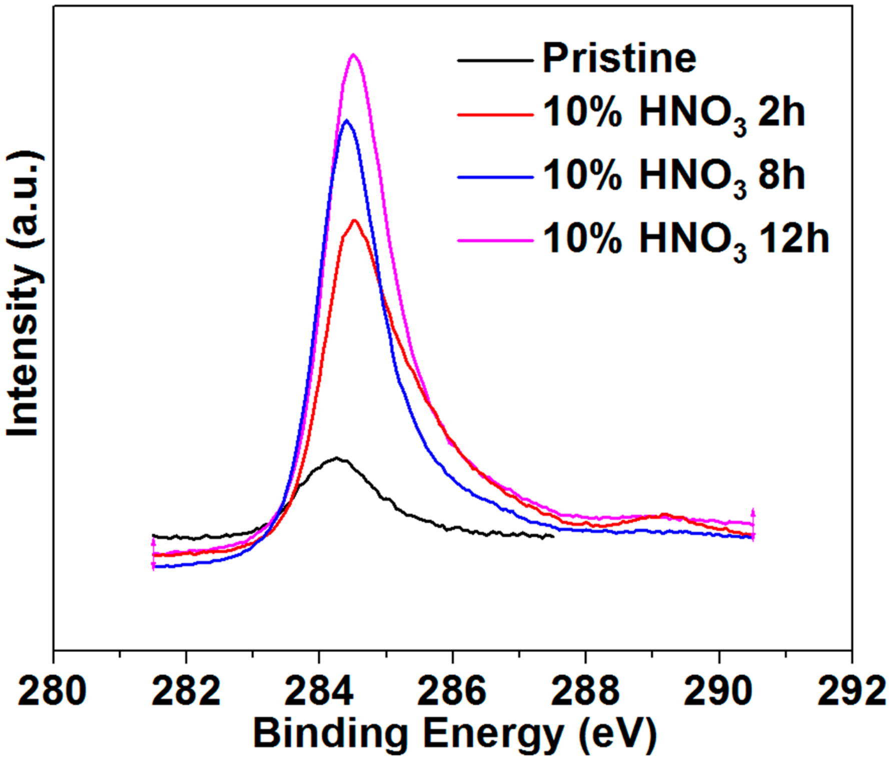

Figure S3. C (1s) XPS scan results of different HNO_3 etching duration samples.
